# How CAR T cells can be shielded from prostaglandin E2 signaling to enhance their therapeutic activity

**DOI:** 10.1016/j.omton.2026.201204

**Published:** 2026-04-27

**Authors:** Janina Dörr, David Andreu-Sanz, Sebastian Kobold

**Affiliations:** 1 Institute of Clinical Pharmacology, LMU University Hospital, LMU Munich, Munich, Germany; 2 German Cancer Consortium (DKTK), Partner Site Munich, a partnership between the DKFZ Heidelberg and the LMU University Hospital, LMU Munich, Munich, Germany; 3 Einheit für Klinische Pharmakologie (EKLiP), Helmholtz Zentrum München - German Research Center for Environmental Health Neuherberg, Munich, Germany; 4 German Center for Lung Research (DZL), partner site Munich, Munich, Germany

## Main text

**Figure 1:**
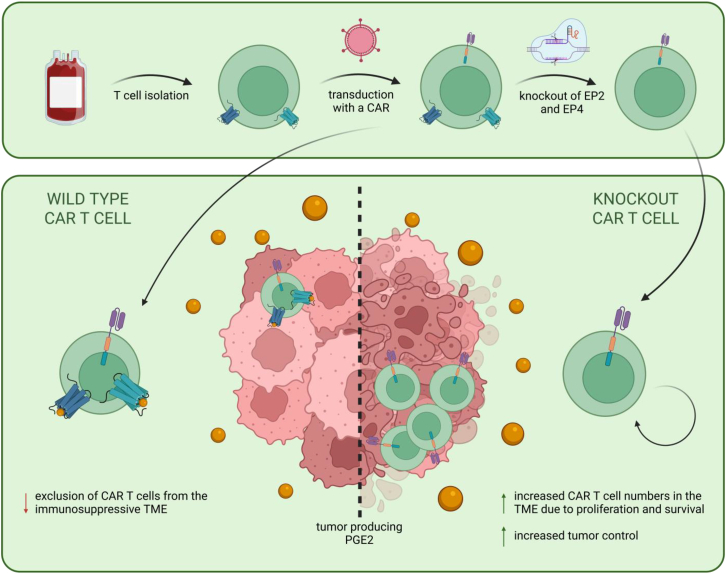
Graphical abstract of dual EP2/EP4 knockout in CAR T cells to overcome PGE2-mediated suppression in solid tumors Top, illustrates our strategy to engineer PGE2-resistant CAR T cells by CRISPR-Cas9-mediated knockout of the PGE2 receptors EP2 and EP4. Conventional CAR T cells (bottom) are restricted in their proliferative capacity and their ability to survive in the PGE2-rich tumor microenvironment, leading to a failure to control the tumor. Dual EP2^−/−^EP4^−/−^ CAR T cells (bottom, right) lack PGE2-responsiveness, maintain robust expansion, and accumulate effectively in PGE2-rich tumors. Key outcomes: rescued proliferation, superior intratumoral persistence, and enhanced tumor control. This modular platform creates “PGE2-resistant” CAR T cells applicable to diverse solid tumor indications. This figure was created using BioRender.

CAR T cells have revolutionized the treatment of hematologic malignancies but continue to face major challenges in solid tumors. One important reason is the profoundly immunosuppressive tumor microenvironment (TME), which restricts T cell function and thereby limits durable tumor control. Among the mediators shaping this environment, prostaglandin E2 (PGE2) is a key immunosuppressive molecule that inhibits a broad range of immune cells, including natural killer (NK) cells^1^ and dendritic cells.^2^ We recently showed that PGE2 is also a potent driver of T cell dysfunction in the TME.^3^ Specifically, we demonstrated that tumor-derived PGE2 downregulates the interleukin 2 (IL-2) receptor on T cells, thereby reducing their proliferative and survival capacities, limiting their abundance and persistence within the tumor and ultimately impairing tumor control. We hypothesized this to be an immunosuppressive mechanism highly relevant for CAR T cell therapy of solid tumors and thus worked on overcoming this barrier. In our recently published manuscript,^4^ we genetically disrupted PGE2 signaling by knocking out its receptors, prostaglandin E2 receptor 2 and 4 (EP2 and EP4) in CAR T cells. We will summarize this strategy and its key outcomes, which are captured schematically in a graphical abstract (Figure 1).

## Generation and characterization of EP2/EP4 knockout CAR T cells

PGE2 mediates its effects largely through the G protein-coupled receptors EP2 and EP4, which signal via cyclic AMP (cAMP) and downstream effectors such as CREB. Because EP2 and EP4 show substantial functional redundancy, we reasoned that single receptor targeting might be insufficient to fully release T cells from PGE2-mediated suppression. We first set out to establish whether abrogation of PGE2 signaling in T cells via CRISPR-Cas9-based simultaneous knockout of EP2 and EP4 was technically feasible and could improve their fitness and anti-tumor activity in a solid tumor setting. We confirmed that double knockout (EP2^−/−^EP4^−/−^) CAR cells completely lacked downstream EP2/EP4 signaling by measuring PGE2-induced cAMP production and CREB phosphorylation. In contrast, single EP2 or EP4 knockout only partially reduced these readouts, indicating residual PGE2 responsiveness and confirming the need for dual targeting to fully block this pathway. In human CAR T cells, we quantified editing efficiencies using next-generation sequencing of the EP2 and EP4 loci. We achieved high knockout rates of approximately 70%–90% for both single and double knockouts, demonstrating that efficient dual editing is feasible within clinically relevant workflows. To address safety and specificity, we performed whole-genome sequencing for one donor and did not detect off-target mutations attributable to our editing strategy.

## *In vitro* functionality in the presence of PGE2

We next examined how EP2/EP4 knockout affects CAR T cell function *in vitro*, focusing in particular on conditions that mimic a PGE2-rich TME. Upon exposure to PGE2, control CAR T cells exhibited markedly reduced proliferation. By contrast, EP2^−/−^EP4^−/−^ CAR T cells proliferated robustly even in the presence of PGE2. When we analyzed *in vitro* tumor control, we found that the superior performance of dual-knockout CAR T cells under PGE2 exposure was primarily driven by rescued proliferation and sustained cell numbers, rather than by per-cell differences in acute activation. Conventional activation markers did not differ between edited and unedited CAR T cells, reinforcing the notion that PGE2 acts predominantly as a brake on T cell expansion and persistence. Removing EP2/EP4 signaling restores these parameters without fundamentally altering activation thresholds.

## *In vivo* efficacy and patient-derived tumor systems

To assess whether the *in vitro* advantages of EP2/EP4-deficient CAR T cells translate into better tumor control *in vivo*, we evaluated our engineered cells in solid tumor xenograft models. In both a model of pancreatic ductal adenocarcinoma and a mesothelioma model, CAR T cells targeting tumor-associated antigens were injected into mice bearing established, PGE2-producing tumors. Dual-knockout CAR T cells achieved improved tumor control and prolonged survival compared with their unedited counterparts. As in the OT-I system, these benefits were closely associated with increased intratumoral persistence of EP2^−/−^EP4^−/−^ CAR T cells. Longitudinal analysis of tumor-infiltrating lymphocytes (TILs) revealed that edited CAR T cells were present at higher frequencies within the tumor, consistent with protection from PGE2-induced loss of proliferative capacity. Despite their enhanced accumulation, we did not observe major differences in exhaustion markers, activation status, or differentiation profiles between EP2^−/−^EP4^−/−^ and control CAR T cells *in vivo*. This suggests that the principal effect of PGE2 resistance is to preserve CAR T cell numbers and persistence, rather than to generate a qualitatively distinct or less exhausted phenotype.

To test our strategy in a more clinically relevant system, we tested EP2^−/−^EP4^−/−^ CAR T cells in patient-derived organotypic tumor spheroids (PDOTS) from multiple solid tumor entities, including pancreatic ductal adenocarcinoma and colorectal cancer. In these *ex vivo* systems that better recapitulate the complexity of the human TME, dual-knockout CAR T cells consistently displayed superior anti-tumor activity compared with unedited cells, supporting the translational relevance of our approach.

## Implications and outlook

Taken together, our work shows that PGE2-EP2/EP4 signaling constitutes a central immunosuppressive axis that constrains CAR T cell expansion and persistence in solid tumors and that dual genetic ablation of EP2 and EP4 effectively neutralizes this barrier. This highlights the potential value of blocking PGE2 signaling for solid tumor therapy, especially in the context of T cell-dependent therapeutics such as CAR T cells.

Historically, the most prevalent strategy for this is the use of COX inhibitors or other non-steroidal anti-inflammatory drugs (NSAIDs), which dampen PGE2 synthesis. Epidemiological and interventional studies suggest that long-term use of NSAIDs can reduce the incidence of several solid tumors.^5^ However, this encouraging preventive signal has not translated into safe and effective therapeutic regimens for established disease.^5^ Sustained systemic COX inhibition is limited by gastrointestinal and cardiovascular toxicities as it does not discriminate between healthy physiological and tumor-promoting functions of PGE2.^6^ Clinical trials of NSAIDs or COX-2 inhibitors alone or in combination with conventional cancer therapies such as chemotherapy or radiotherapy have therefore not yielded broadly applicable anti-tumor treatments. However, recently several trials were started in combination with T cell-targeting immunotherapy. A retrospective study looking at the concurrent COX inhibitor use during immune checkpoint inhibition therapies in metastatic melanoma and non-small cell lung cancer indicates a clinical benefit of this combination in both cancer entities.^7^ To reduce side effects caused by systemic COX inhibition, more selective pharmacological antagonists of EP receptors are currently under development. Several EP4 or EP2 and EP4 dual-inhibitors showed a favorable safety profile in early phase clinical trials, also in combination with immune checkpoint inhibition (ICI). However, data on their efficacy is currently still limited.^8^^,^^9^^,^^10^ To this date, there are no clinical trials combining PGE2-inhibition with CAR T cell therapy, although first preclinical efforts are being made.^11^

Our work offers an alternative solution by confining PGE2 pathway modulation to the therapeutic immune cells rather than the whole organism. By selectively deleting EP2 and EP4 in CAR T cells, we render the cell product resistant to PGE2-mediated suppression without altering PGE2 levels or signaling in other tissues. This cell-intrinsic strategy circumvents many of the systemic safety liabilities that have hampered COX inhibition and other pharmacological approaches. By isolating CAR T cells from PGE2, we were able to preserve proliferative and survival signals in a hostile TME. This results in higher intratumoral CAR T cell abundance and improved tumor control in both mouse models and patient-derived samples, without inducing overt hyperactivation or exacerbated exhaustion. More broadly, our data position EP2^−/−^EP4^−/−^ CAR T cells as a modular, PGE2-resistant therapeutic platform that can, in principle, be combined with diverse CAR specificities and tumor indications. Because the intervention is restricted to the cell product, it avoids systemic pharmacological blockade of PGE2 and its related substantial safety concerns.^6^

Future studies will be needed to integrate EP2/EP4 editing into clinical manufacturing pipelines, examine long-term safety and persistence and explore how this strategy interacts with standard therapies and other immunomodulatory agents. Our findings support the concept that blockade of PGE2 signaling can help unlock the full potential of CAR T cell therapy for solid tumors.

## Acknowledgments

This study was supported by the Bavarian Cancer Research Center (BZKF) (TANGO to S.K.), the Deutsche Forschungsgemeinschaft (DFG, grant number: KO5055-2-1 and KO5055/3-1 to S.K.), the international doctoral program “i-Target: immunotargeting of cancer” (funded by the Elite Network of Bavaria; to S.K.), the Melanoma Research Alliance (grant number 409510 to S.K.), Marie Sklodowska-Curie Training Network for Optimizing Adoptive T Cell Therapy of Cancer (funded by the Horizon 2020 programme of the European Union; grant 955575 to S.K.), Marie Sklodowska-Curie Training Network for tracking and controlling therapeutic immune cells in cancer (funded by the Horizon Programme of The EU, grant 101168810 to S.K.), Else Kröner-Fresenius-Stiftung (IOLIN to S.K.), German Cancer Aid (DEFEAT PDAC, AvantCAR.de and grant number 70117182 to S.K.), the Wilhelm-Sander-Stiftung (to S.K.), Ernst Jung Stiftung (to S.K.), Institutional Strategy LMUexcellent of LMU Munich (within the framework of the German Excellence Initiative; to S.K.), the Go-Bio-Initiative (to S.K.), the m4-Award of the Bavarian Ministry for Economical Affairs (to S.K.), Bundesministerium für Bildung und Forschung (to S.K.), European Research Council (starting grant 756017, PoC grant 101100460 and CoG 101124203 to S.K.), by the SFB- TRR 338/3 2026–452881907 (to S.K.), Fritz-Bender Foundation (to S.K.), Deutsche José Carreras Leukämie Stiftung (to S.K.), Hector Foundation (to S.K.), Bavarian Research Foundation (BAYCELLATOR to S.K.), the Monika-Kutzner Foundation (to S.K.), the Bruno and Helene Jöster Foundation (360° CAR to S.K.), the Dr. Rurainski-Foundation (to S.K.) and the Constanze and Dr. Brigitte Wegener Foundation. J. D. received funding supporting this study from the Deutsche Gesellschaft für Immun-und Targeted Therapie. The figure was created using BioRender.

## Declaration of interests

S.K. has received honoraria from Plectonic, TCR2 Inc., Regeneron, Miltenyi, Galapagos, Cymab, Novartis, BMS, and GSK. He is an inventor of several patents in the field of immuno-oncology. S.K. received license fees from TCR2 Inc and Carina Biotech and he also received research support from TCR2 Inc., Tabby Therapeutics, Catalym GmbH, Plectonic GmbH, and Arcus Bioscience for work unrelated to the manuscript.
